# Supplementary figures and images for: Evaluation of the Effect of Storage Methods on Fecal, Saliva, and Skin Microbiome Composition
Source: mSystems. 2021 Apr 27;6(2):e01329-20. doi: 10.1128/mSystems.01329-20 (PMC8092129; doi:10.1128/mSystems.01329-20)

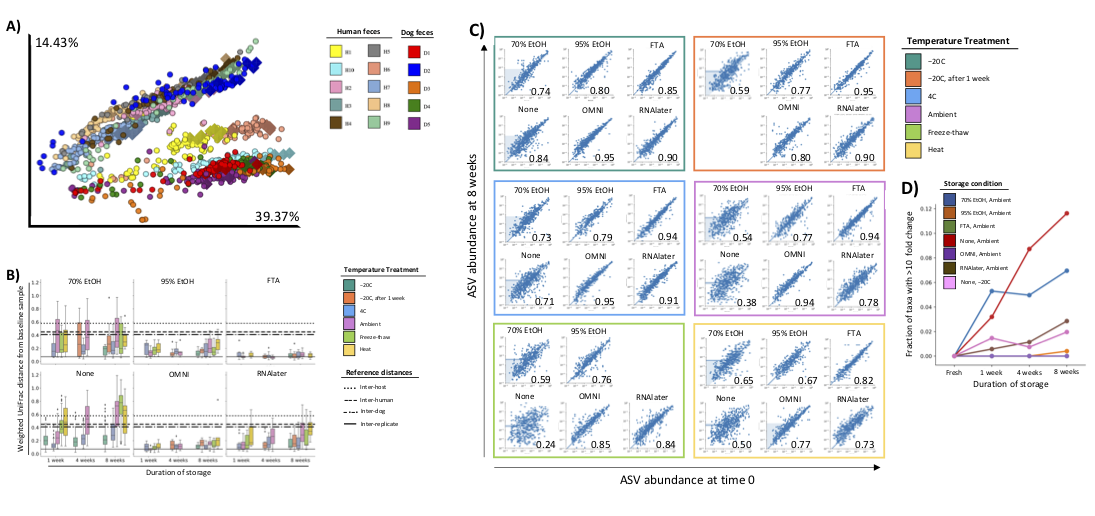

Supplement: FIG S1 [file mSystems.01329-20-sf001.tif]

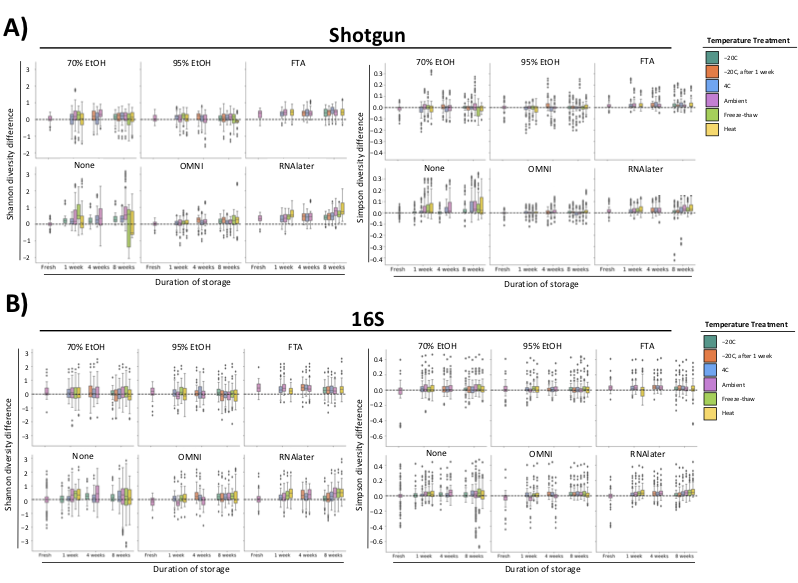

Supplement: FIG S2 [file mSystems.01329-20-sf002.tif]

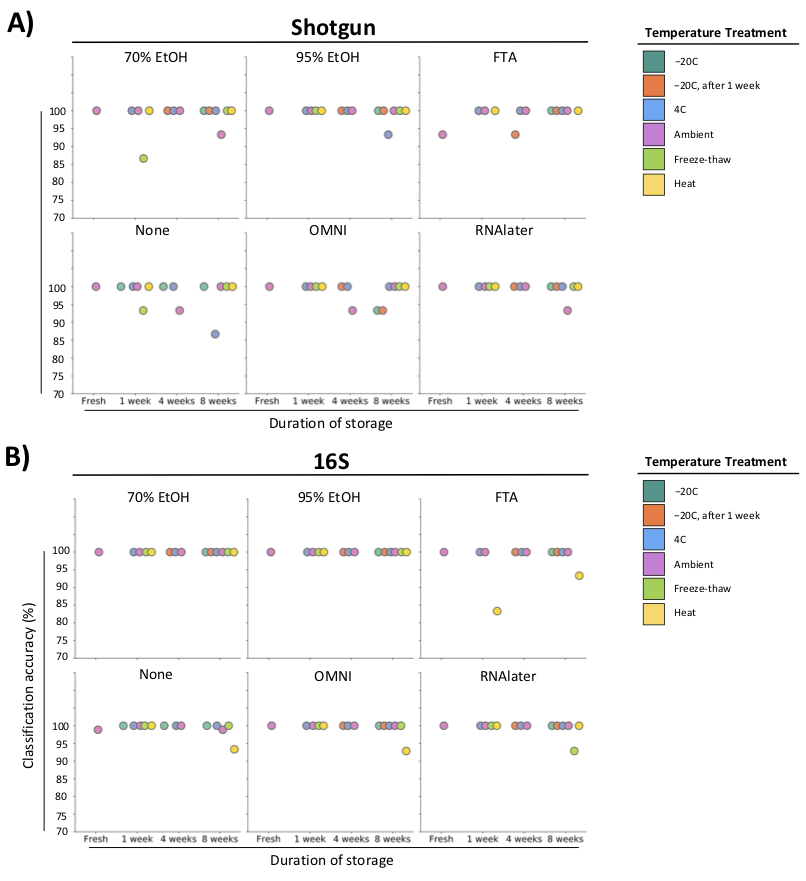

Supplement: FIG S3 [file mSystems.01329-20-sf003.tif]

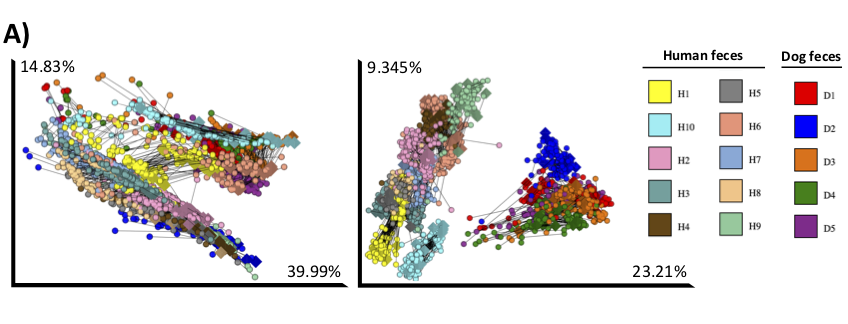

Supplement: FIG S4 [file mSystems.01329-20-sf004.tif]
